# Human Flt3L Generates Dendritic Cells from Canine Peripheral Blood Precursors: Implications for a Dog Glioma Clinical Trial

**DOI:** 10.1371/journal.pone.0011074

**Published:** 2010-06-11

**Authors:** Weidong Xiong, Marianela Candolfi, Chunyan Liu, A. K. M. Ghulam Muhammad, Kader Yagiz, Mariana Puntel, Peter F. Moore, Julie Avalos, John D. Young, Dorothy Khan, Randy Donelson, G. Elizabeth Pluhar, John R. Ohlfest, Kolja Wawrowsky, Pedro R. Lowenstein, Maria G. Castro

**Affiliations:** 1 Gene Therapeutics Research Institute, Cedars-Sinai Medical Center and Department of Medicine and Department of Molecular and Medical Pharmacology, David Geffen School of Medicine, University of California Los Angeles, Los Angeles, California, United States of America; 2 Pathology, Microbiology, and Immunology, School of Veterinary Medicine, University of California Davis, Davis, California, United States of America; 3 Department of Comparative Medicine, Cedars-Sinai Medical Center, Los Angeles, California, United States of America; 4 Department of Pediatrics, University of Minnesota, Minneapolis, Minnesota, United States of America; 5 Department of Small Animal Clinical Sciences, University of Minnesota, St. Paul, Minnesota, United States of America; 6 Department of Neurosurgery, University of Minnesota, Minneapolis, Minnesota, United States of America; 7 Department of Medicine, Cedars-Sinai Medical Center and David Geffen School of Medicine, University of California Los Angeles, Los Angeles, California, United States of America; The University of Chicago, United States of America

## Abstract

**Background:**

Glioblastoma multiforme (GBM) is the most common primary brain tumor in adults and carries a dismal prognosis. We have developed a conditional cytotoxic/immunotherapeutic approach using adenoviral vectors (Ads) encoding the immunostimulatory cytokine, human soluble *fms*-like tyrosine kinase 3 ligand (hsFlt3L) and the conditional cytotoxic molecule, i.e., Herpes Simplex Type 1- thymide kinase (TK). This therapy triggers an anti-tumor immune response that leads to tumor regression and anti-tumor immunological memory in intracranial rodent cancer models. We aim to test the efficacy of this immunotherapy in dogs bearing spontaneous GBM. In view of the controversy regarding the effect of human cytokines on dog immune cells, and considering that the efficacy of this treatment depends on hsFlt3L-stimulated dendritic cells (DCs), in the present work we tested the ability of Ad-encoded hsFlt3L to generate DCs from dog peripheral blood and compared its effects with canine IL-4 and GM-CSF.

**Methodology/Principal Findings:**

Our results demonstrate that hsFlT3L expressed form an Ad vector, generated DCs from peripheral blood cultures with very similar morphological and phenotypic characteristics to canine IL-4 and GM-CSF-cultured DCs. These include phagocytic activity and expression of CD11c, MHCII, CD80 and CD14. Maturation of DCs cultured under both conditions resulted in increased secretion of IL-6, TNF-α and IFN-γ. Importantly, hsFlt3L-derived antigen presenting cells showed allostimulatory potential highlighting their ability to present antigen to T cells and elicit their proliferation.

**Conclusions/Significance:**

These results demonstrate that **hs**Flt3L induces the proliferation of canine DCs and support its use in upcoming clinical trials for canine GBM. Our data further support the translation of **hs**Flt3L to be used for dendritic cells' vaccination and gene therapeutic approaches from rodent models to canine patients and its future implementation in human clinical trials.

## Introduction

Glioblastoma multiforme (GBM) is the most common and aggressive primary brain tumor in adults. GBM accounts for 35% of all primary brain tumor cases and 80% of malignant brain tumors (www.cbtrus.org). The standard of care for treatment of GBM, which consists of surgical resection, followed by radiation therapy and chemotherapy with temozolomide, leads to a median survival of 6–18 months [1]. Thus, novel approaches are being developed for the treatment of this devastating disease [2]–[6]. Our previous studies have shown that a combined conditional cytotoxic-immunotherapy approach using adenoviral vectors (Ad) that express the immunostimulatory cytokine human soluble *fms*-like tyrosine kinase 3 ligand (hsFlt3L, Ad-hsFlt3L), and the conditionally cytotoxic Herpes Simplex Type 1- thymide kinase (TK, Ad-TK) induces tumor regression, long-term survival, and immunological memory in rats and mice bearing large intracranial syngeneic glioblastomas or intracranial melanomas [7]–[10]. Intratumoral expression of Ad-hsFlt3L recruits dendritic cells into the brain parenchyma [11], improving tumor antigen presentation, while Ad-TK exerts a cytotoxic effect exclusively in proliferating GBM cells in the presence of ganciclovir (GCV), leading to the release of tumor antigens and inflammatory molecules from dying tumor cells [7], [8], [10].

Spontaneous canine GBM constitutes an attractive animal model for testing novel therapies for GBM. GBM is the most common primary brain tumor in dogs, specially in certain breeds, such as Boston terriers and Boxers [11]–[16]. Spontaneous GBM in dogs exhibit the same histopathological features, clinical signs and standard treatment as the human disease [17]–[20]. A clinical trial in canine GBM patients would provide very useful data: i) due to the infiltrative nature of spontaneous GBM in dogs, they will allow testing the efficacy of novel therapies, and their toxicity to the normal brain, ii) due to the large size of the dog brain, they would be useful for assessing the doses and volumes needed in order to optimize treatment protocols, iii) the fact that dogs undergo similar standard care for GBM allows performing clinical trials that mimic more closely the clinical scenario, in which new therapies are implemented in conjunction with standard of care, iv) the individual variability of outbreed dogs better mimics the clinical scenario. Thus we aim to test the efficacy of Ad-hsFlt3L and Ad-TK in dog patients bearing spontaneous GBM. To this end we and others have previously demonstrated the feasibility of delivering therapeutic transgenes to dog GBM cells *in vitro* and dog brain cells *in vivo* upon intracranial injection of gene therapy vectors, such as type 5 adenoviral vectors [21]–[23] and adeno-associated viral vectors [24]. Also, we have shown that TK expression exerts a powerful cytotoxic effect on dog GBM cells [21], [22]. However, the functionality of **human soluble** Flt3L on dog dendritic cells (DCs) has been controversial [25]–[27]. Considering that the efficacy of this treatment depends on an anti-tumor immune response triggered by dendritic cells upon Flt3L stimulation [10], and as a prelude for a clinical trial in canine GBM patients, we aimed to evaluate whether hsFlt3L expressed by the therapeutic Ad-hsFlt3L would exert a trophic effect on dog dendritic cells.

It has been previously demonstrated that DCs can be successfully obtained from dog peripheral blood mononuclear cells (PBMC) using canine IL-4 and GM-CSF, *in vitro* [28]. In order to evaluate the ability of hsFlt3L to generate DCs from dog PBMC we compared it with canine IL-4 and GM-CSF. Our results demonstrate that hsFLT3L is capable of generating DCs from PBMC cultures with very similar characteristics to canine IL-4 and GM-CSF-cultured DCs. Thus, indicating that hsFlt3L is able to modulate the function of dog dendritic cells. We found that dendritic cells generated using hsFlt3L express monocytic and dendritic cell markers, they are capable of phagocytosis and overexpression of activation markers and pro-inflammatory cytokines upon maturation. Also, they are effective at antigen presentation to T cells. Thus, this report supports the use hsFlt3L encoded within an Ad in combination with Ad-TK (plus GCV) in dog GBM patients. Our data will be critical for translating the use of hsFlt3L in both dendritic cells' vaccination approaches and also in gene therapeutic strategies from rodents to canine patients and ultimately to human patients.

## Results

### Morphological features of DCs cultured with hsFlt3L or canine IL-4 and GM-CSF

**Figure 1:**
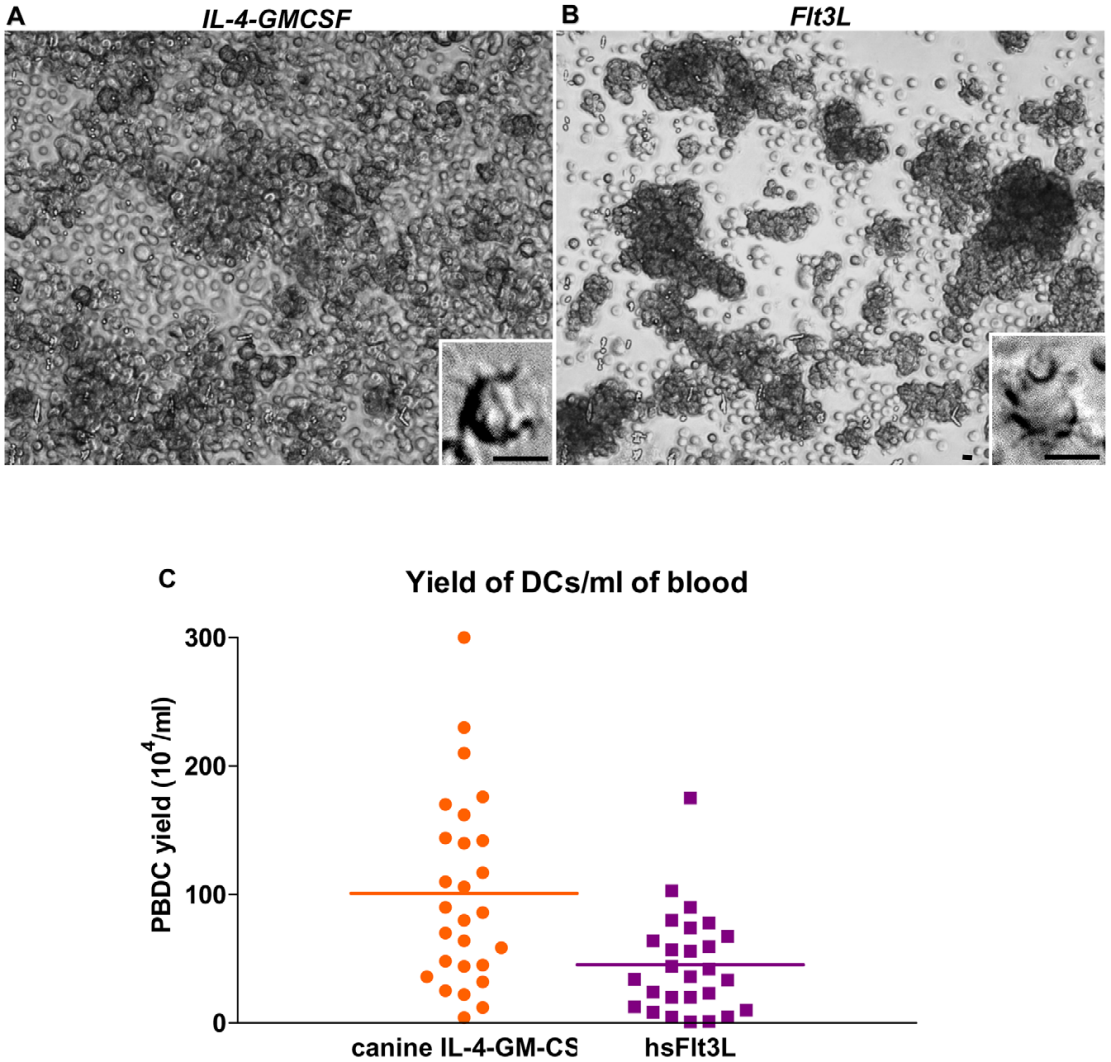
Morphology of peripheral blood-derived dog dendritic cells cultured with canine IL-4+GM-CSF or Ad.hFlt3L conditioned medium. White cells from dog peripheral blood were purified using Lymphocyte Separation Media and cultured in the presence of recombinant canine IL-4 (50 ng/ml) and GM-CSF (33 ng/ml) or Ad.hsFlt3L conditioned medium (10 ng/ml). The images show the appearance of the culture after 7 days in culture. Low magnification images (20×) show typical spheres formed in culture supplemented with canine IL-4+GM-CSF (**A**) or hsFlt3L (**B**). Insets show higher magnification images of cells that exhibit typical long cytoplasmic processes characteristic of dendritic cell morphology. Scale bars: 10 µm. **C**, After 7–9 days in culture non-adherent cells were collected and counted. The graph shows the yield of cells per ml of blood obtained from 27 dogs after culture with hsFlt3L or canine-IL-4/GM-CSF.

Adherent dog peripheral blood leucocytes were cultured with hsFlt3L derived from Ad-hsFlt3L conditioned medium or with canine IL-4 and GM-CSF. Although the gross appearance of both cultures was similar and adherent cellular aggregates were readily observed after 4–6 days of culture in both conditions, the amount of cells outside the aggregates was higher in the presence of canine-IL-4 and GM-CSF (Fig. 1). After 1 week, cultured cells that differentiated into non-adherent cells exhibiting thin cytoplasmic processes were observed in hsFlt3L as well as in IL-4+GM-CSF cultures (Inset Fig. 1). At day 7–9 floating cells were collected and cellular aggregates were dislodged for flow cytometric analysis of immune cell markers or to activate the immature DCs. The average yield of cells collected from these cultures (n = 27 dogs) was 45×10^4^ (±7.5×10^4^) and 101×10^4^ (±14×10^4^) for hsFlt3L and canine-IL-4/GM-CSF cultures, respectively (Fig. 1C).

### Phenotypic characterization of canine DCs cultured in the presence hsFlt3L or canine IL-4 and GM-CSF

**Figure 2:**
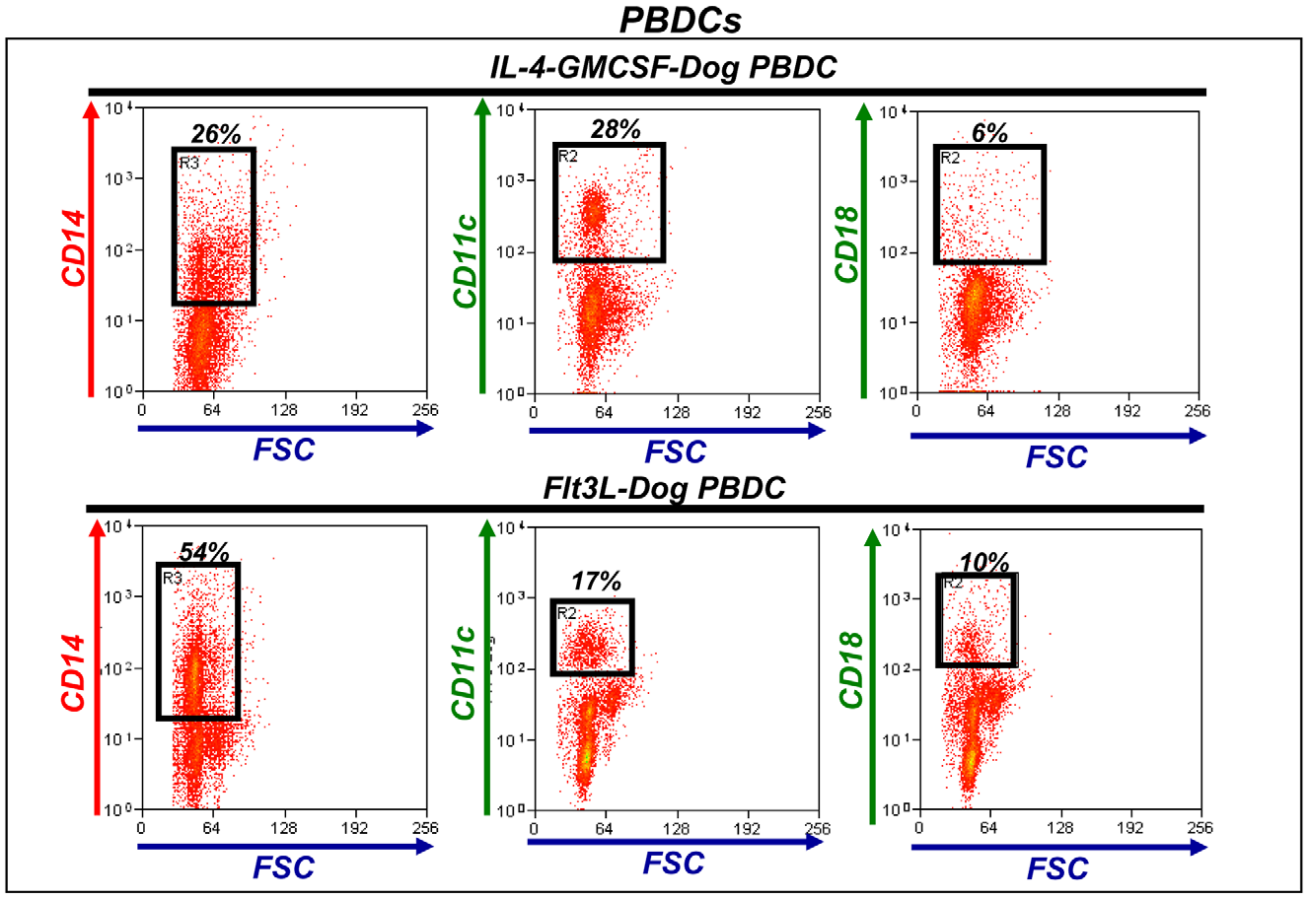
Phenotypic characterization of dog peripheral blood DC cultures. Peripheral blood cultures were collected after 7 days of culture with recombinant canine IL-4 and GM-CSF or Ad.hFlt3L conditioned medium. Immune cell populations in the culture were identified using antibodies against CD14 (monocytes), CD11c (dendritic cells) and CD18 (macrophages). Numbers in representative dot plots indicate the percentage of positive cells. Approximately 12,000 events were collected from each sample. Dot plots are representative of 4 dogs. SSC: Side Scatter; FSC: Forward Scatter.

In order to determine the ability of hdFlt3L to generate DCs from of peripheral blood precursors, we assessed the expression of DC markers. We studied the phenotype of peripheral blood DC cultures 1 week after incubation in the presence of hsFlt3L or canine IL-4 and GM-CSF. In peripheral blood cultures incubated with hsFlt3L, we found that around half of the non-adherent cells expressed the monocytic marker CD14 (Fig. 2). Expression of DC marker CD11c and macrophage CD18 was observed in 20% and 10% of cells, respectively. These results were comparable with peripheral blood cultures incubated in the presence of IL-4 and GM-CSF (Fig. 2).

### Activation status of canine DCs cultured in the presence of hsFlt3L or canine IL-4 and GM-CSF

**Figure 3:**
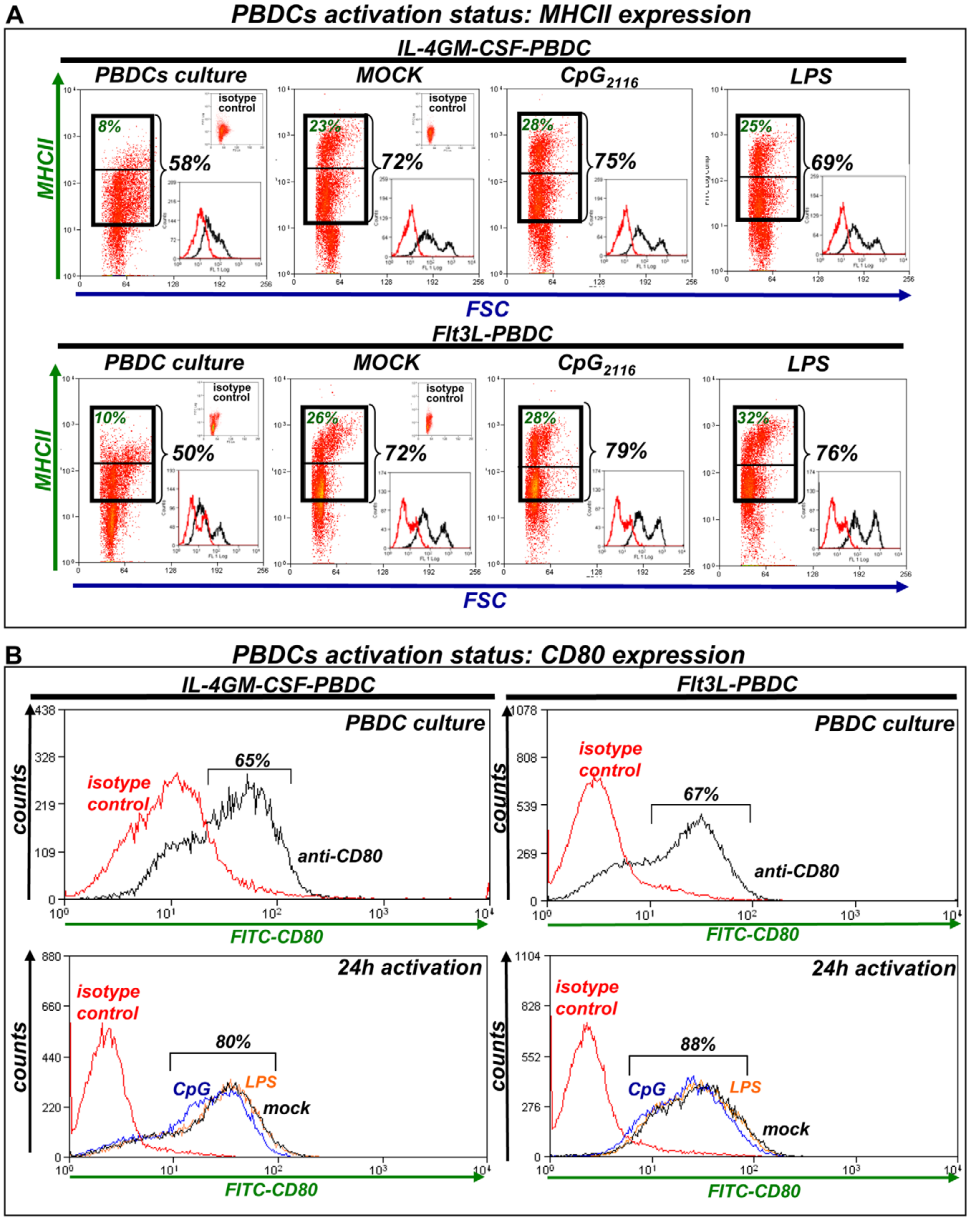
Activation status of dog peripheral blood DC cultures. Peripheral blood cultures were collected after 7 days in culture (PBDC culture). Cells were then incubated for additional 24 h without added cytokines in the absence (mock) or presence of CpG_2216_ or LPS. Activation status was assessed by flow cytometry using an antibody against Major Histocompatibility Complex type II (MHCII, **A**) or against the costimulatory marker CD80 (**B**). Black numbers in representative dot plots or histograms indicate the percentage of MHCII^+^ or CD80^+^ cells. Green numbers indicate MHCII^high^ cells. Insets on the top right show the dot plot of cells incubated with isotype control mouse IgG1. Insets on the bottom right show representative MHCII expression histograms (black) and their respective isotype control (red). Approximately 12,000 (**A**) or 30,000 events (**B**) were collected from each sample. Dot plots are representative of 3–4 dogs. SSC: Side Scatter; FSC: Forward Scatter.

**Figure 4:**
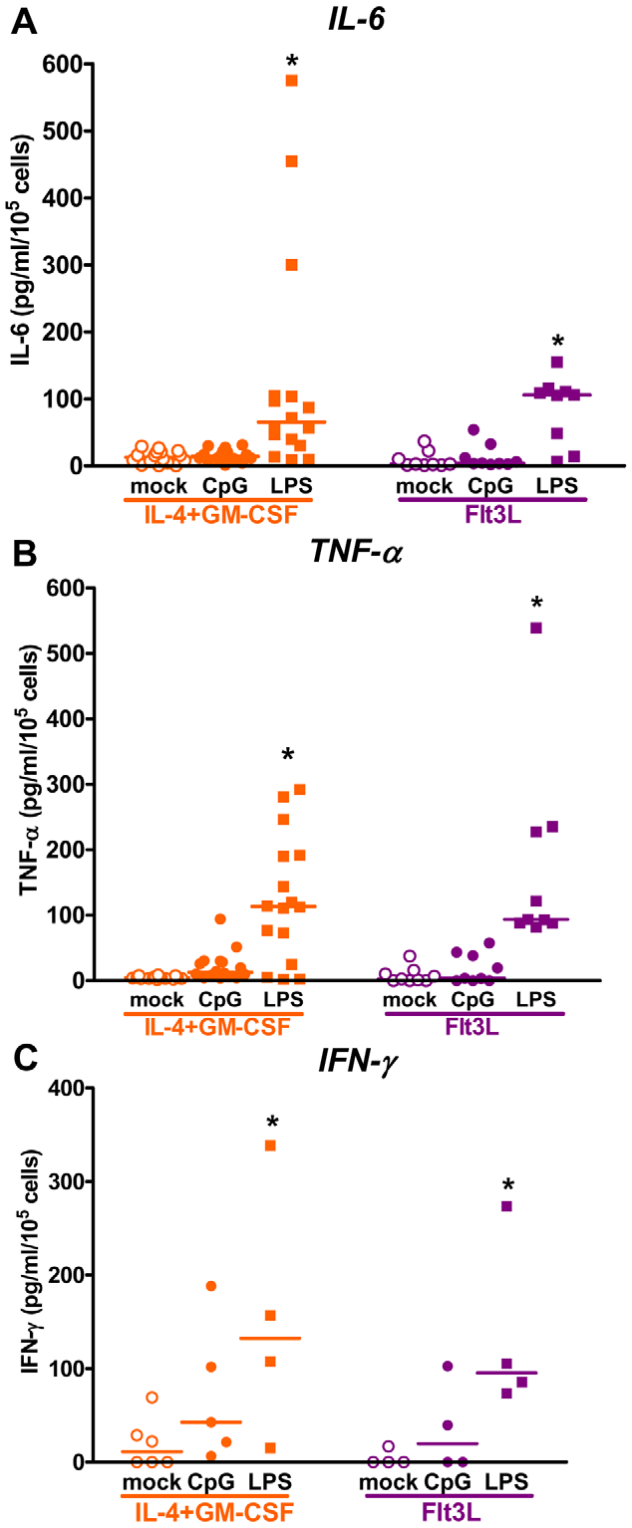
Cytokine release from dog PBDC cultures. Peripheral blood cultures were collected after 7 days in culture with recombinant canine IL-4 and GM-CSF, or Ad.hFlt3L conditioned medium and incubated for additional 24 h without added cytokines in the absence (mock) or presence of CpG_2216_ or LPS. Release of IL-6 (**A**), TNF-α (**B**) and IFN-γ (**C**) was determined by ELISA. *p<0.05 vs mock (Two-way ANOVA followed by Tukey's Multiple Comparison test). Scatter plots show values from individual dog blood cultures (n = 4–12).

Optimal antigen presentation requires maturation of DCs; this is crucial to mount an anti-tumor immune response. Thus, we evaluated the activation status of peripheral blood DC cultures incubated with hsFlt3L or canine IL-4/GM-CSF. After 7 days in culture 40–50% of cells in both peripheral blood cultures expressed intermediate levels of MHCII, while ∼10% expressed high levels of MHCII (Fig. 3 A). In addition ∼65% cells in both cultures expressed the co-activation marker CD80 (Fig. 3 B). In order to induce maturation of DCs, cells were collected 7 days after culture, cytokines were removed and cells were incubated with ODN CpG or LPS for 24h. Activation led to over expression of MHCII in hsFlt3L- and IL-4/GM-CSF-derived DCs under the three conditions tested (cytokine withdrawal +/− CpG or LPS). MHCII expression was observed in over 70% of the cells, half of which were MHCII^high^ (Fig. 3 A) and CD80 expression was detected in ∼80–90% of the cells, regardless the activation stimulus (Fig. 3 B). We also measured the release of cytokines into the cell culture supernatant during 24 h activation (Fig. 4). We found that LPS strongly stimulated the release of IL-6 and TNF-α from hsFlt3L- and IL-4/GM-CSF-derived canine DCs, while CpG increased only slightly the levels of IL-6 and TNF-α (Fig. 4 A and B). On the other hand, both CpG and LPS increased the release of IFN-γ from hs-Flt3L and IL-4/GM-CSF-cultured canine DCs (Fig. 4 C). Taken together these results suggest that the maturation of DCs generated from peripheral blood with hsFlt3L is comparable to IL-4/GM-CSF. Also, there were no differences in the sensitivity of both types of cultures to TLR agonists.

### Phagocytic activity of canine DCs cultured with hsFlt3L or canine IL-4 and GM-CSF

**Figure 5:**
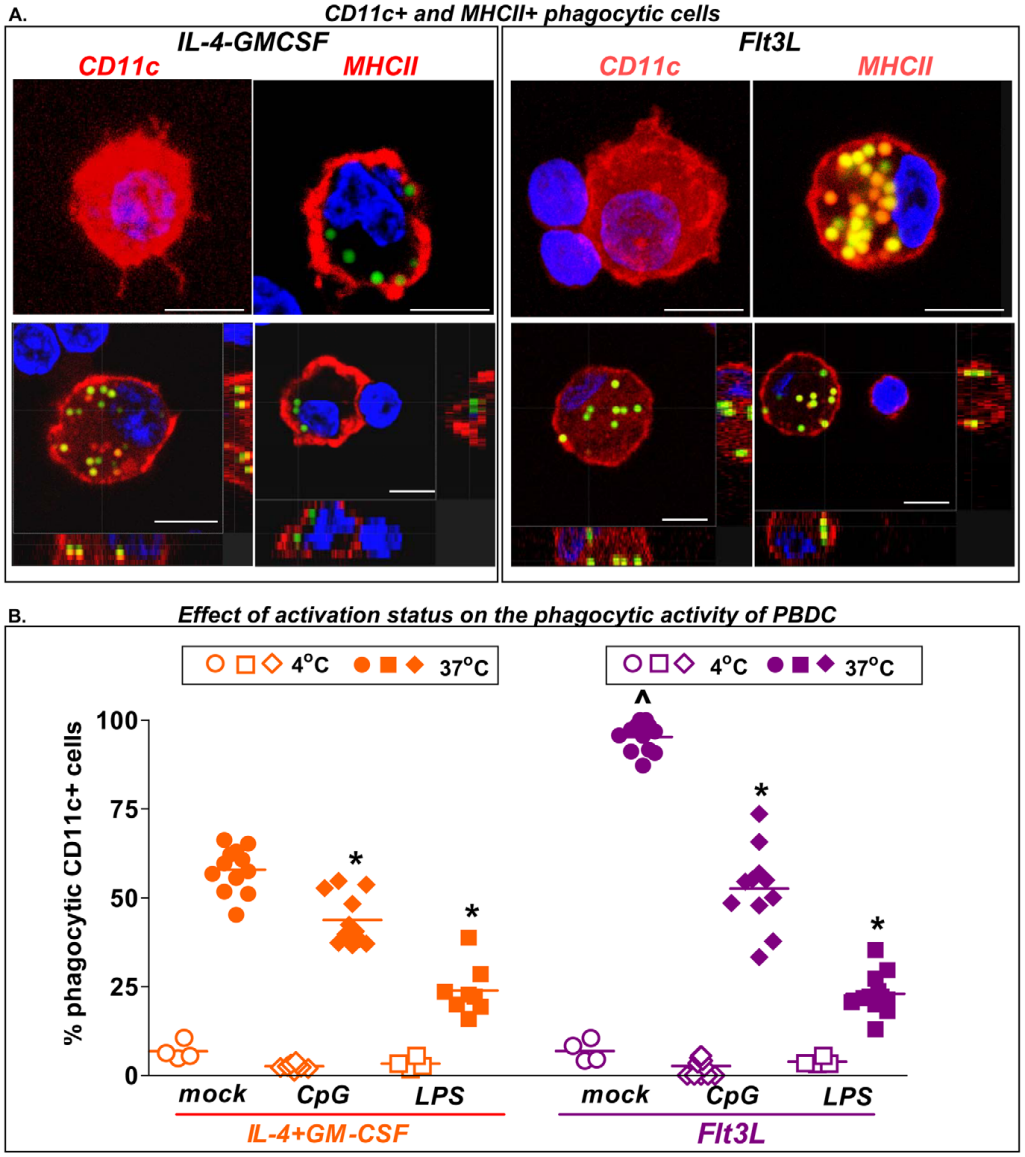
DC phagocytic activity. Peripheral blood cultures were collected after 7 days in culture with recombinant canine IL-4 and GM-CSF or Ad.hFlt3L conditioned medium and incubated for additional 24 h without added cytokines in the absence (mock) or presence of CpG_2216_ or LPS. Activated dog DCs were mixed with 1.0 µm yellow-green fluorescent FluoSpheres for 4 hrs at 4°C or 37°C. **A**, Confocal microphotographs show CD11c^+^ and MHCII^+^ cells (red) containing yellow-green beads in their cytoplasm. Nuclei were stained with DAPI (blue). Scale bars: 10 µm. **B**, Scatter plot shows the quantification of CD11c^+^ DCs containing yellow-green fluorescent FluoSpheres in their cytoplasm. *p<0.05 vs mock ∧ p<0.05 vs. IL-4/GM-CSF (Two-way ANOVA followed by Tukey's Multiple Comparison test).

Considering that tumor antigen uptake by DCs is a crucial event in the initiation of the anti-tumor immune response induced by the combined Ad-hsFlt3L+Ad-TK therapy [10], we evaluated the phagocytic activity of peripheral blood-derived dendritic cells (PBDCs) generated with hsFlt3L. We compared the phagocytic activity of hsFlt3L- or IL-4/GM-CSF-derived PBDCs to take up yellow green fluorescence beads. After 2h incubation with beads, cells were fixed and stained by immunofluorescence with anti-MHCII or anti-CD11c antibodies. MHCII^+^ cells and CD11c^+^ DCs containing beads in their cytoplasm were readily detected in hsFlt3L and IL-4/GM-CSF cultures analyzed by confocal microscopy (Fig. 5A). We quantified the phagocytic activity of PBDCs after activation with CpG or LPS. We found that the basal percentage of CD11c^+^ DCs with phagocytic activity was higher in cultures derived from hsFlt3L than canine IL-4/GM-CSF (Fig. 5 B). Also, the phagocytic activity of both hsFlt3L- and IL-4/GM-CSF-derived DCs was reduced upon activation with CpG or LPS (Fig. 5 B).

### Human soluble Flt3L enhances the capacity of DCs to induce proliferation of allogeneic T cells

**Figure 6:**
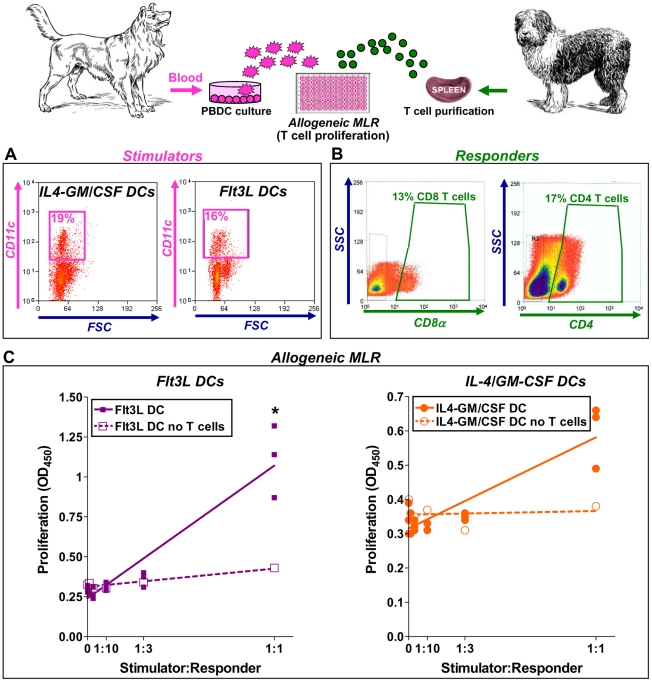
Allogeneic mixed-lymphocyte reaction (MLR). DCs (stimulators, **A**) from dog peripheral blood monocytes cultured with either Ad.hFlt3L conditioned media (purple dots) or IL-4+GM-CSF (orange dots) were incubated with allogeneic spleen T cells (responders, **B**) purified by Fluorescence Activated Cell Sorting (FACS) from the spleen of a second dog. Dot plots show the percentage of CD11c^+^-cells (**A**) and the percentage of CD8^+^ and CD4^+^ T cells collected from dog spleen by FACS (**B**). SSC: Side Scatter; FSC: Forward Scatter. **C**, T cell proliferation was determined by BrdU incorporation after 4 days in co-culture. Additional controls included DCs incubated without T cells (broken lines). *p<0.05 vs. DCs no T cells (Randomization test).

We observed that hsFlt3L can generate DCs from peripheral blood precursors; activation stimulates the release of Th1 cytokines and reduces the phagocytic activity of DCs. Finally, since the ability to prime and stimulate T cell proliferation is an important assessment of the functionality of DCs [25], we performed a mixed leucocyte reaction (MLR) to determine the proliferation of allogeneic lymphocytes induced by mature DCs. DCs were generated from dog peripheral blood, cultured with hsFlt3L or canine IL-4/GM-CSF for 1 week, and activated for additional 24h with CpG (Fig. 6 A). Splenocytes were collected from a second dog and CD4^+^and CD8α^+^ T cells were purified by flow cytometry cell sorting after staining with specific antibodies (Fig. 6 B). Dog T cells were co-cultured with increasing numbers of allogeneic mature DCs for 7 days. T cell proliferation was assessed by incorporation of BrdU. As controls, DCs were incubated in the absence of T cells. We found that increasing numbers of hsFlt3L-derived PBDCs exerted a stimulatory effect on the proliferation of allogeneic T cells (Fig. 6 C). Although at a lesser extent, canine IL-4/GM-CSF-derived dog DCs also induced allogeneic T cell proliferation. These results indicate that hsFlt3L exerts a potent trophic effect on dog DCs, which in turn efficiently activate proliferation of allogeneic naïve T cells.

## Discussion

We show that human Flt3L expressed from an adenoviral vector generates dendritic cells from dog peripheral blood precursors. Although previous reports have shown that maturation of DCs can be achieved by culturing peripheral blood leucocytes with T cell conditioned media [29] and canine cytokines [28], [30], [31], the ability of human cytokines to exert trophic effects on canine immune cells has been a matter of debate. Weber et al. reported no activity of human TNF-α in the growth of canine DCs and little cross-reaction of human Flt3L with canine immune cells [25]. However, Hagglund et al. demonstrated that while the combination of human GM-CSF, human TNF-α and human IL-4 failed to stimulate proliferation of canine DCs, replacement of human IL-4 by human Flt3L in the cytokine cocktail led to generation of DCs *in vitro* from dog bone marrow progenitors [26]. Moreover, dogs that received daily subcutaneous injections of human recombinant Flt3L for 10 days exhibited increased numbers of leucocytes, mainly monocytes, in peripheral blood and circulating DCs displayed strong allostimulatory potential [27]. Our results show that hsFlt3L exerts a trophic effect on dog DCs that is comparable to the canine cytokine cocktail comprising of IL-4 and GM-CSF. These findings support the rationale of administering an Ad vector encoding hsFlt3L to dog patients bearing spontaneous gliomas to mount an immune response against the tumor. We have previously demonstrated that an immunotherapeutic approach that combines the cytotoxic effect of Ad-TK with the immune stimulation induced by hsFlt3L leads to brain tumor regression and immunological memory in several intracranial rodent tumor models [8], [10], [32].

After 7 days of incubation with hsFlt3L or canine IL-4/GM-CSF, PBMCs cultures contained ∼20–30% CD11c^+^ DCs. Tallying with previous reports, canine IL-4/GM-CSF led to differentiation of peripheral blood precursors into immature DCs, which produced low levels of cytokines and most of the cells displayed phagocytic activity [28], [30], [31]. Our findings suggest that the maturation status of hsFlt3L-derived DCs might be slightly different than IL-4-GMCSF-derived DCs. Phagocytic activity was higher when PBMC were cultured in the presence of hsFlt3L, suggesting that these DCs are less mature than IL-4/GM-CSF-derived DCs [25], [29], [30]. Although activation of both cultures led to increased release of Th1-cytokines, the allostimulatory activity of mature DCs was higher when PBMCs were cultured with hsFlt3L, suggesting an enhanced expression of co-activation molecules. In fact, it has been shown that blocking of CD86 using neutralizing antibodies completely inhibits the stimulatory effect of dog DCs on allogeneic leucocytes [30].

After 7 days of incubation with hsFlt3L or canine IL-4/GM-CSF, PBMCs cultures contained ∼30–50% CD14^+^ monocytes. Although in rodents and humans the expression of CD14 has been shown to be downregulated in DCs upon maturation, it has been proposed that dog DCs derived from PBMC sustain their levels of CD14 [29], [30]. The sustained expression of CD14 in our PBMC cultures incubated with both hsFlt3L and IL-4/GM-CSF was in correlation with the robust stimulatory effect of LPS on TNF-α, IL-6 and IFN- γ release. Accordingly, LPS strongly inhibited the phagocytic activity of DCs derived from both cultures, suggestive of a more mature phenotype [25], [29], [30].

While LPS led to maturation of DCs from both cultures, i.e. hsFlt3L and canine IL-4/GM-CSF, we found little or no effect of CpGs on cytokine release from PBMC-derived DCs. This findings are in accordance with previous results that showed no stimulatory effect of CpGs on proliferation of PBMCs [33] and antigen presentation from dog bone marrow-derived DCs [25]. Although expression of TLR9 was detected in dog PBMCs [34], an array of 11 CpGs only induced a minor stimulatory effect on IFN-γ and IL-12p40 expression in canine PBMCs, and did not affect the expression of IL-12p35, IL-18 and IL-4 [35]. Weak secretory responses to CpG in canine blood derived monocyte-macrophages were attributed to low expression of TLR9 in these cells [36]. Additionally, it has been proposed that canine immune cells exhibit a weaker response to TLR9 agonists than other species [37].

It is important to note that, in agreement with other authors [30], we observed widespread phenotypic variations between dogs throughout the study, which included over 20 dogs. This variability is an important feature of dog patients for cancer research and the development and testing of novel therapeutics, since they better resemble the variability encountered in the clinical scenario.

In conclusion, our results demonstrate that **hs**Flt3L successfully modulates the function of canine DCs. These findings support the translation of hsFlt3L to be used in combination with Ad-TK for gene therapy applications or in dendritic cell vaccination protocols from rodent glioma models to canine GBM patients to eventually being implemented in human patients.

## Materials and Methods

### Ethical Statement

All animal work was conducted according to the NIH guidelines and was approved by the Institutional Care and Use Committee (IACUC #2183) at Cedars Sinai Medical Center.

### Adenoviral vectors

Ad-hsFlt3L used for this study is a first-generation replication-defective recombinant adenovirus type 5 vector expressing human soluble Flt3L under the transcriptional control of the human cytomegalovirus intermediate early promoter within the E1 region. Ad-hsFlt3L was generated as previously described [38], [39]. Titration was carried out by end-point dilution, cytopathic effect assay, with centrifugation of infected 96-well plates as described in detail by Nyberg-Hoffman et al [40]. The titer for Ad-hsFlt3L was 1.32∼2.64×10^12^ iu/ml. Viral preparation was free of replication-competent adenovirus (RCA) and LPS contamination, as assessed by the Limulus amebocyte gel clot assay (Biowhittaker, UK) [41], [42]. All relevant adenoviral generation methods and quality control procedures are described in detail in Southgate et al [39].

### Generation of human soluble Flt3L conditioned media

5×10^6^ COS-7 cells were seeded in a T-75 flask and infected with Ad-hsFlt3L virus at 500 iu/cell in RPMI (CellGro) containing 10% heat-inactivated fetal calf serum, 2 mM L-glutamine, 1% MEM nonessential amminoacids, 100 IU/ml penicillin and 100 µg/ml streptomycin). The supernatant was harvested 72 h later and centrifuged to discard cellular debris. HsFlt3L conditioned media was stored at −80°C. The concentration of Flt3L in the conditioned media was determined by ELISA following manufacturer's instructions (R&D System, Quantikine ELISA kit, *Cat # DFK00*).

### Dog blood and splenocytes collection

The protocol to collect peripheral blood from dogs was approved by the Institutional Care and Use Committee (IACUC) at Cedars Sinai Medical Center. Peripheral blood was collected by venous puncture under appropriate manual restraint from a total of 27 random-bred dogs housed at Comparative Medicine Department, Cedars Sinai Medical Center. Twenty ml of blood were collected from each dog into BD Vacutainer® K2 EDTA Plus Blood Collection Tubes (BD Biosciences, *Cat. # 366643*). In addition, 1 inch^3^ blocks of spleen were collected under surgical anesthesia from one dog following an IACUC-approved non-survival surgical procedure.

### DC generation using hsFlt3L or canine IL-4/GM-CSF

Dog peripheral blood leucocytes were obtained by gradient centrifugation using lymphocyte separation medium (LSM, density 1.077–1.080 g/ml, Cellgro® Mediatech, *Inc. Cat. No 25-072-CV*) following the manufacturer's instructions. Leucocytes were resuspended in RPMI 1640 containing 10% heat-inactivated fetal calf serum, 2 mM L-glutamine, 100 IU/ml penicillin and 100 µg/ml streptomycin. Peripheral blood (PB) leucocytes were incubated in 6-well plates (1.2×10^7^ cells/well, FALCON®, *Cat # 353046*) for 2 h at 37°C in 2 ml supplemented RPMI. After 2 h, about 30% of the cells had already attached. Non-adherent leucocytes were removed by repeated washes with phosphate buffered saline (PBS). Adherent PBMCs were then cultured with 2 ml culture medium containing hsFlt3L conditioned medium (final concentration of hsFlt3L: 10 ng/ml). As positive controls, adherent PBMCs were cultured in RPMI containing 50 ng/ml recombinant canine IL-4 (R&D Systems, *Cat # 754-CL*) plus 33 ng/ml recombinant canine GM-CSF (R&D Systems, *Cat # 1546-GM*). Half of the culture medium was replaced with fresh medium containing cytokines every 2∼3 days. Non-adherent cells were harvested 7–10 days later and analyzed by flow cytometry or seeded in 24-well plates (∼0.5–1×10^6^/well in 0.2 ml) to induce DC maturation. These cells were incubated for additional 24h without cytokines and in the presence of 5 µg/ml LPS (InvivoGen, *Cat # tlrl-eklps*) or 50 µg/ml ODN CpG2007 (Cell Sciences Hycult biotechnology, *cat # HC4038*) or ODN CpG 2216 (Alexis Biochemicals *cat #TALX-746-005*) to induce maturation of DCs. Supernatant was collected for detection of Th1 cytokine release and cells were harvested for flow cytometric analysis, phagocytosis assays or allogeneic MLR.

### Flow cytometry

Flow cytometric analysis was performed on peripheral blood-derived dendritic cells (PBDCs) collected from cultures expanded in the presence of hsFlt3L or canine IL-4/GM-CSF, or on PBDCs incubated for additional 24 h in the absence of cytokines and with LPS or CpG. 75,000–1,000,000 cells were used for staining, depending on the yield of the culture. Cells were stained for 30 min with 50 µl RPE-conjugated mouse anti-human CD14 (1∶5, ABD Serotec, cat # *MCA1568PE*) or RPE-conjugated mouse IgG2a (ABD Serotec, *cat # MCA929PE*) as isotype control. Also, cells were incubated with 50µl mouse anti-canine CD11c, MHCII, CD80 and CD18 (8 µg/ml, generated in the Department of Veterinary, Pathology, Microbiology, and Immunology, School of Veterinary Medicine, University of California, Davis, CA) or mouse IgG1 (eBiosciences, *cat # 14-4714-82*) as isotype control. Cells were washed and incubated with 50 µl FITC-conjugated goat anti-mouse 488 (1∶400, Molecular Probes, *cat # A-21121*) for additional 30 min. Finally, cells were washed and resuspended in PBS with 1%FBS. Flow cytometric analysis was performed using a FACScan flow cytometer (Becton Dickinson). Data were analyzed using DAKO Summit v4.3 software.

### Phagocytosis assessment

24 h after treatment with LPS or CpG in the absence of cytokines (see above), PBDCs were seeded in 24-well plate and mixed with 1.0 µm yellow-green fluorescent FluoSpheres (30 beads per cell, Invitrogen, Molecular Probes, *Cat # F8823*). After 4 h at 37°C or 4°C as control, cells were harvested, washed and fixed with 4% PFA. Cells were then stained with 8 µg/ml mouse anti-canine CD11c and MHCII or mouse IgG1 isotype control (all described above). Cells were washed and incubated with goat anti-mouse Alexa 594 (1∶400, Molecular Probes, *cat # A11032*). Nuclei were stained with DAPI (5 µg/ml, Molecular Probes) and mounted with ProLong Antifade (Molecular Probes). Confocal micrographs were obtained using a Leica confocal microscope TCS SP2 with AOBS equipped with a 405-nm violet-diode UV laser, 488-nm argon laser, and 594- and 633-nm helium-neon lasers; using a HCX PL APO 63 3 1.4 numerical aperture oil objective (Leica Microsystems Heidelberg, Mannheim, Germany).

### Allogeneic mixed leukocyte reaction (MLR)

After 7 days culture in hsFlt3L or canine IL-4/GM-CSF, non-adherent PBDCs were harvested and incubated 6 h with 50 µg/ml CpG_2007_ to be used as stimulators in an alloegeneic MLR. Dog spleen was harvested as described above from a second dog and 1 inch^3^ cubes were dissected and homogenized to release splenocytes. Red blood cells were removed by incubating in 3 volumes of ACK solution (0.15 mM NH_4_Cl, 10 mM KHCO_3_, and 0.1 mM sodium EDTA at pH 7.2) for 2 min and then washed with supplemented RPMI. Cells were passed trough a cell strainer BD Pharmingen 35-2350) to discard large debris. Splenocytes were frozen in 10% DMSO-90% FBS in liquid nitrogen until used for the allogeneic MLR. Dog splenocytes were thawed and stained with 8 µg/ml mouse anti-canine CD4 and CD8 (generated in the Department of Veterinary, Pathology, Microbiology, and Immunology, School of Veterinary Medicine, University of California, Davis, CA). Cells were washed and incubated with FITC-conjugated goat anti-mouse 488 (1∶400) for additional 30 min. CD4^+^ and C8^+^ T cells were then sorted by FACS (Becton Dickinson). 50,000 total responder T cells (50% CD4 and 50% CD8) were incubated in flat bottom 96-well plates (BECTON DICKINSON *cat #353072*) with increasing numbers of allogeneic stimulator DCs (0, 1∶100, 1∶30, 1∶10, 1∶3, 1∶1) in 100 µl supplemented RPMI at 37°C. DCs were incubated in the absence of T cells as controls. Four days later 10 µl BrdU labeling solution (1∶100) was added to the media for additional 18 h and BrdU incorporation into all genomic cellular DNA strands was assessed by ELISA following manufacturer's instructions (Roche-Applied-Science, *Cat # 11 647 229 001*). Absorbance of the samples was measured without stop solution in an ELISA reader (Spectramax Plus, Molecular Devices) at 370 nm.

### ELISA

Release of IL-6, TNF-α and IFN-γ was determined by ELISA following the manufacturer's protocol (R&D system, Quantikine ELISA kit *cat# CA6000, CATA00* and *EL781*, respectively). Absorbance was read on a 96-well plate reader (Spectramax Plus, Molecular Devices) at 450 nm and 570 nm to subtract background.

### Statistical Analysis

NCSS Statistical and power analysis software, (Kaysville, Utah, USA) was employed to perform the statistical analysis. MLR curves were analyzed using randomization test. The phagocytosis and ELISA data were analyzed by ANOVA. When data failed normality or Levene's test for Variance Homogeneity, they were log-transformed. Differences between groups were considered significant at p<0.05.
